# Spontaneous Cervical Epidural Hematoma Following COVID-19 Illness Presenting to a Chiropractor: A Case Report

**DOI:** 10.7759/cureus.32199

**Published:** 2022-12-05

**Authors:** Eric C Chu, Robert J Trager, Colin R Lai, John S Shum

**Affiliations:** 1 Integrative/Complementary Medicine, New York Chiropractic and Physiotherapy Centre, Kowloon, HKG; 2 Chiropractic, Connor Whole Health, University Hospitals Cleveland Medical Center, Cleveland, USA; 3 Chiropractic, Logan University, Chesterfield, USA; 4 Chiropractic, New York Chiropractic and Physiotherapy Centre, Kowloon, HKG; 5 Radiology, Hong Kong Advanced Imaging, Kowloon, HKG

**Keywords:** cervical spine, covid-19, neck pain, spinal epidural hematoma, chiropractic

## Abstract

Cervical epidural hematoma (CEH) is a rare and potentially fatal condition in which blood accumulates in the epidural space of the cervical spine.

A 64-year-old man presented to a chiropractor with a two-week history of sudden-onset neck pain, shoulder pain, occipital headache, and numbness in the shoulders and upper extremities. He had recovered from a mild course of coronavirus disease 2019 (COVID-19) illness one month prior. The patient’s primary care provider had previously prescribed a nonsteroidal anti-inflammatory drug for his neck pain. However, his symptoms worsened, and he visited the emergency department where he had unremarkable cervical spine radiographs and was discharged with a diagnosis of neck strain. The chiropractor ordered cervical spine magnetic resonance imaging (MRI), revealing a ventral CEH extending from C2 to C5. The chiropractor referred the patient to a nearby hospital for urgent management. The patient was admitted and observed, progressively improved, and did not require surgery. After 10 weeks in the hospital the patient was asymptomatic, a follow-up MRI revealed resolution of the CEH, and the patient was discharged.

While the current case highlights a temporal relationship between COVID-19 and CEH, further research is needed to determine if COVID-19 is a risk factor for this condition. Clinicians who encounter patients with spinal disorders must be able to recognize the clinical features of CEH and refer these patients for emergency care and/or neurosurgical evaluation.

## Introduction

Cervical epidural hematoma (CEH) is a form of spinal epidural hematoma (SEH), a condition in which blood accumulates in the spinal epidural space. It often presents with acute onset neck pain with radiation into the extremities with or without neurologic deficit [1]. In some cases, CEH may be spontaneous, occurring without any trauma [1,2]. A thorough understanding of SEH is particularly important for providers that manage spine-related disorders, such as chiropractors, given the potential morbidity of unrecognized SEH, which rarely may result in death [1,2].

Spontaneous SEH is extremely rare, with an estimated incidence of 0.1 patients per 100,000 per year, and mostly affects the thoracic spine [3]. Spontaneous SEH typically occurs in the fourth to fifth decades of life and has a slight male predominance [1,2]. Risk factors for SEH include trauma, spine surgery, anticoagulant use, arteriovenous malformations, vertebral hemangioma, coagulopathy, and possibly hypertension [1,2]. Spinal manipulative therapy, a common treatment utilized by chiropractors, has also been suggested as a potential trigger for SEH [4].

In addition, recent case reports have suggested that SEH may develop as a complication of coronavirus disease 2019 (COVID-19) [5-8]. While research relating SEH to COVID-19 is limited, recent systematic reviews have suggested that several bleeding conditions such as intracranial hemorrhage and muscle hematomas are also potential complications of COVID-19 [9,10]. While this phenomenon could be the result of the illness itself, for example, due to coagulation abnormalities, endothelial dysfunction, and thrombosis [9], bleeding disorders may also arise due to medical treatments for COVID-19 that have anticoagulant properties [10].

Chiropractors are portal-of-entry healthcare providers who often manage spinal complaints such as low back and neck pain [11], and rarely encounter patients with severe vascular pathology. One study reported that chiropractors in the United States encounter a patient with a severe life-threatening condition about once every 2.5 years in practice, on average [12]. However, the frequency at which patients with SEH present to chiropractors is unclear. Post a literature search of PubMed, Google Scholar, and the Index to Chiropractic Literature, using the search terms “chiropractic” and “chiropractor” and “epidural hematoma” and hand-searching a recent review paper [13], on November 18, 2022, we were unable to identify any published cases in which SEH or CEH had been identified by a chiropractor.

This case describes a patient who developed neck pain after recovery from COVID-19 and was ultimately diagnosed with CEH after visiting a chiropractor.

## Case presentation

A 64-year-old man presented to a chiropractor in a multidisciplinary office with a two-week history of sudden-onset neck pain, bilateral shoulder pain, and occipital headache. He reported the mean severity of his neck pain and headache was an eight out of 10 on an 11-point numeric pain rating scale. He also described intermittent shoulder numbness and reported that pain and numbness occasionally radiated diffusely into the upper extremities, hands, and fingers. He denied any visual disturbances, dizziness, photophobia, nausea, vomiting, bowel/bladder, or sleep disturbances, but endorsed a recent course of COVID-19 illness. The patient denied a history of trauma and had no history of anticoagulant or antiplatelet medication use, cardiovascular disease, or hypertension. He had been retired for four years from his occupation as a bus driver, was a non-smoker, and was a social drinker.

The patient’s course of acute COVID-19 was relatively uncomplicated. He tested positive for COVID-19 five weeks prior to his presentation at the chiropractic office in April 2022. Testing was performed at a Hong Kong Community Testing Centre maintained by the Department of Health, via nasopharyngeal swab and polymerase chain reaction testing, and was analyzed in an accredited laboratory. During his course of COVID-19, he had mild symptoms of cough and sinus congestion and briefly had a fever. He was quarantined at home for 10 days and did not need to visit the hospital, take supplemental oxygen, or any antiviral or other specific medications for COVID-19. He took acetaminophen over the counter for symptom relief and felt that he had fully recovered.

Two weeks preceding his chiropractic visit, his family physician initially treated his neck pain with the nonsteroidal anti-inflammatory drug diclofenac. However, the pain worsened over a week which prompted him to visit the emergency department. There, he received cervical spine radiographs, which were interpreted as normal, and he was discharged with a diagnosis of cervical sprain/strain. Cervical spine computed tomography (CT) and magnetic resonance imaging (MRI) was not performed at the emergency department. Given his worsening symptoms, which were unresponsive to treatment, he presented to a chiropractor for a second opinion.

Upon initial evaluation by the chiropractor, the patient was noted to have a guarded neck posture and straight neck with a limited, painful active range of motion in all directions. The passive cervical range of motion was painful and limited to 30° of extension (normal ≥ 50°), 50° of rotation bilaterally (normal ≥ 80°), and 45° of flexion (normal ≥ 90°). Attempted passive flexion also exacerbated the patient’s neck pain and occipital headache. The chiropractor identified hypertonicity and tenderness in the bilateral upper trapezii, sternocleidomastoids, right rhomboid, and right levator scapulae muscles. The chiropractor also noted motion restriction and tenderness at C5/6, C7/T1, and T1/2 via palpation. The maximal cervical compression test provoked the patient’s neck and ipsilateral shoulder pain when performed on each side. An upper limb tension test did not exacerbate the patient’s symptoms on either side. Valsalva testing and cervical distraction likewise did not alter the patient’s symptoms. Neurologic examination including cranial nerve tests, motor and sensory tests, muscle stretch reflexes, Hoffman and Babinski reflex tests, Romberg testing, and observation of the patient’s gait revealed no abnormalities.

The chiropractor initially considered cervical disc herniation as the initial working diagnosis given the acute onset of neck pain with radiation. However, given the patient’s unusual presentation with a broad and bilateral subjective sensory distribution, as well as grossly limited active and passive range of motion, the chiropractor withheld treatment and instead arranged for a cervical spine MRI without contrast within the facility’s imaging center. The chiropractor considered that spinal manipulation could aggravate the patient’s symptoms given his limited cervical spine mobility, apparent muscle guarding, and potential neural involvement.

**Figure 1:**
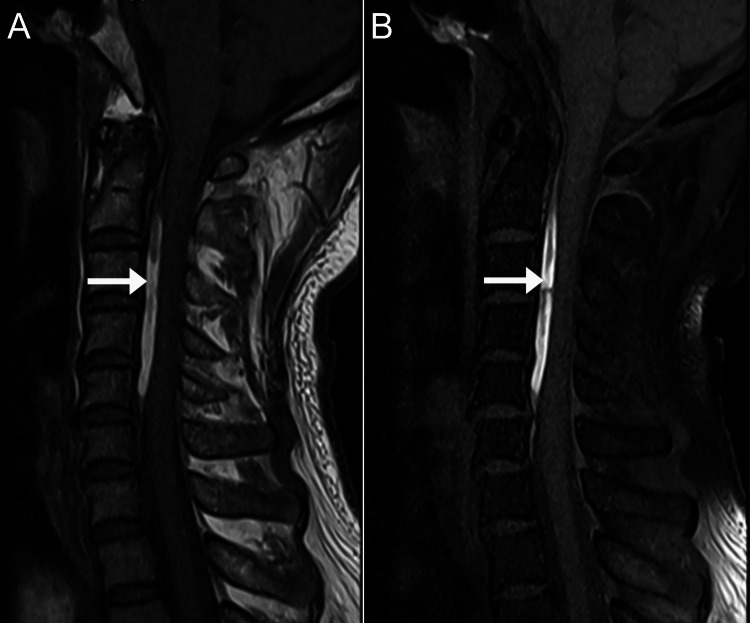
Magnetic resonance imaging of the cervical spine, mid-sagittal T1-weighted images A: Shows a linear hyperintense layer in the anterior epidural space spanning the vertebral levels C2 to C5 measuring 1.2 x 0.4 x 5.4 cm (arrow). B: On a fat-suppressed sequence, this layer remains hyperintense (arrow), which suggests there is no macroscopic epidural fat within this layer.

**Figure 2:**
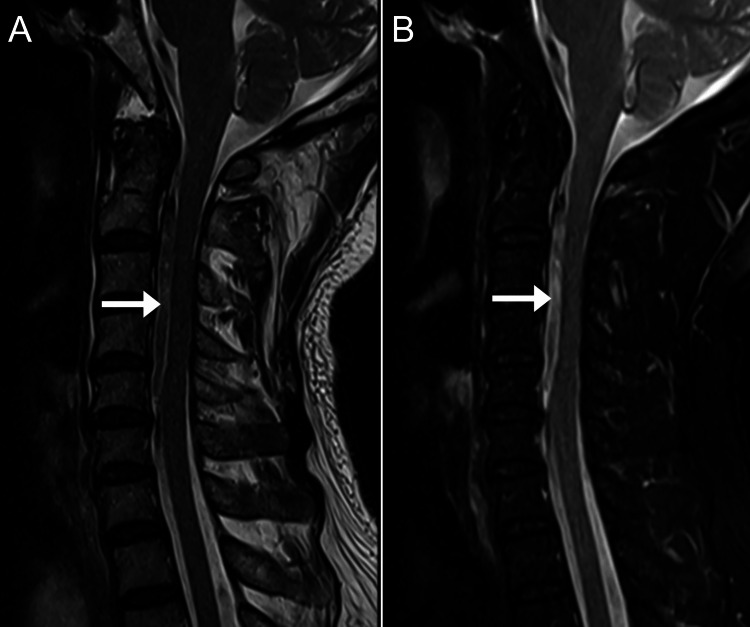
Magnetic resonance imaging of the cervical spine, mid-sagittal T2-weighted images A: A heterogeneously hyperintense and hypointense signal layer in the anterior epidural space (arrow). B: In the T2 fat-suppressed sequence, this layer is mildly hyperintense (arrow).

**Figure 3:**
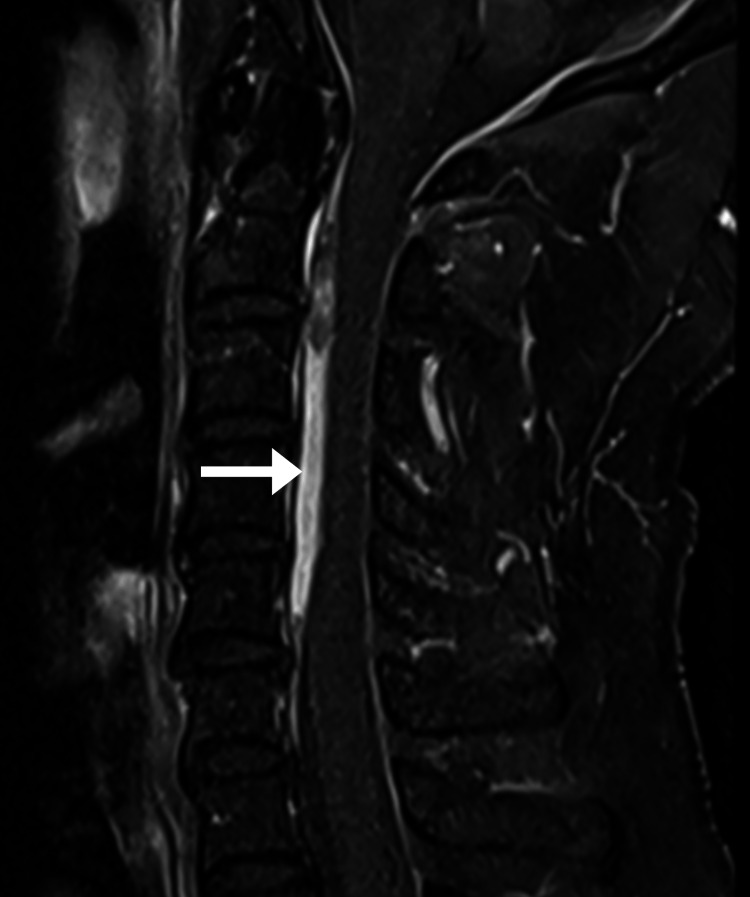
Cervical magnetic resonance imaging, mid-sagittal T1 fat-suppressed sequence with contrast The anterior epidural layer is re-demonstrated (arrow). However, there is no significant contrast enhancement noted, and no suspicious paraspinal soft tissue mass.

**Figure 4:**
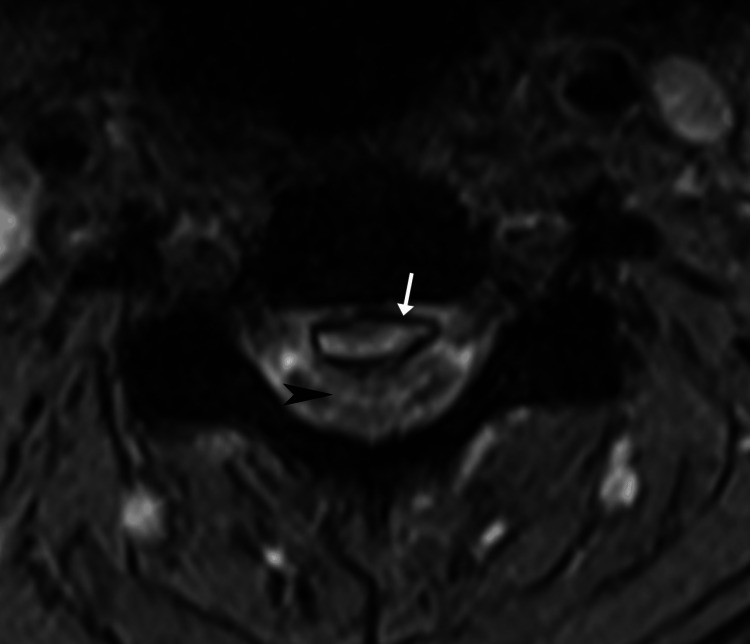
Cervical magnetic resonance imaging, T2-weighted axial at the level of C3/4 The epidural layer is bordered by a T2-hypointense rim (arrow), suggestive of hemosiderin deposition. The spinal cord is markedly impressed, and cord edema is present (black arrowhead).

Four days after the initial chiropractic consultation, the patient underwent a cervical spine MRI. After the non-contrast sequences were obtained, the on-staff radiologist noted an abnormal epidural lesion and recommended the MRI be repeated with gadolinium-based contrast to further differentiate this finding, and this was conducted the same day. The MRI revealed a large lesion in the epidural space of the anterior spinal canal extending from the C2 to C5 vertebral levels without contrast enhancement (Figures 1-3). Mild spinal cord compression was also noted, with edema apparent within the cord (Figure 4). There were no signs of arteriovenous malformation, vertebral hemangioma, or pathologic paraspinal or vertebral edema.

The chiropractor reviewed the MRI and discussed the findings with the radiology team. Cervical epidural hematoma became the working diagnosis while epidural abscess and tumor were also considered. The chiropractor then reviewed the patient’s case and imaging with the clinic’s affiliate neurosurgeon over the phone, who suggested that the patient’s clinical presentation and imaging features were most consistent with CEH, and recommended the patient receive emergency care. The chiropractor then urgently referred the patient to a nearby hospital, where he was admitted for observation.

After admission to the hospital, the patient underwent further workup including laboratory testing which reportedly ruled out the possibility of an epidural abscess. The patient was initially placed on bed rest as a precaution given his CEH. The patient and his family provided regular updates regarding his status and progress via phone or electronic communication. Two days after his admission to the hospital, the patient developed mild paresthesia in his legs. However, his symptoms did not progress further, and he remained neurologically intact and did not develop any gait disturbance. Subsequently, the patient gradually improved. He had not been administered any steroids or other medications and due to his improvement, was deemed to not require surgery or other procedures.

**Figure 5:**
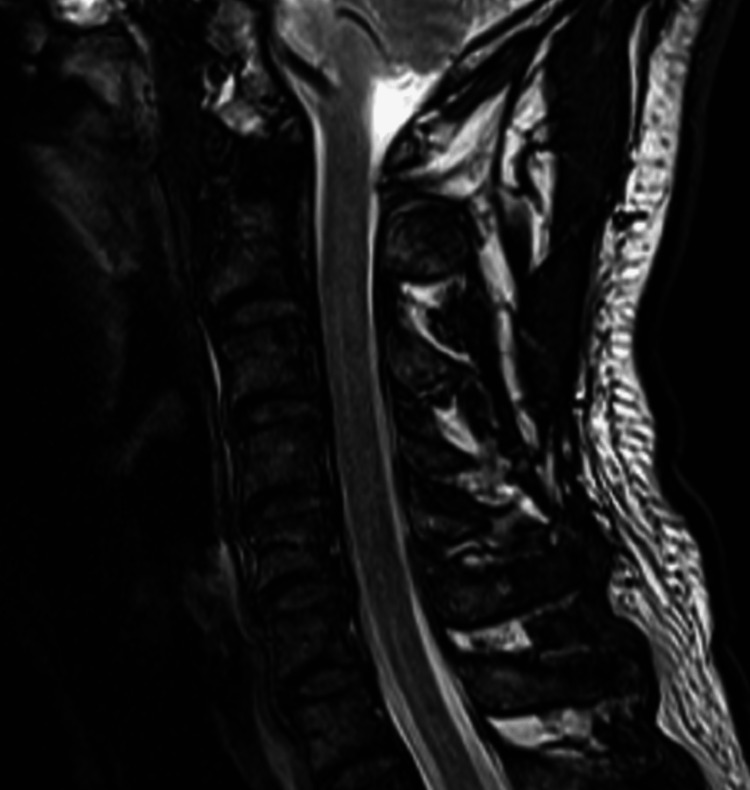
Resolution of the cervical epidural hematoma This mid-sagittal, T2-weighted turbo spin-echo magnetic resonance image taken at the end of the 10th week of the patient’s hospitalization reveals no sign of the previously identified hematoma. Other views (not shown) likewise did not demonstrate any residual hematoma.

By the 10th week of his hospital stay, the patient was completely asymptomatic. The surgical team recommended a repeat cervical spine MRI to evaluate the progression of the CEH. As the MRI revealed that the CEH had fully resolved (Figure 5), the patient was discharged. The patient provided written consent for the publication of this case report and any accompanying images.

## Discussion

The current case illustrates a patient with spontaneous CEH that occurred closely following a COVID-19 illness. While the patient had no overt neurologic deficits (i.e., hyperreflexia, hypoesthesia, weakness), he did have severe pain, a broad and bilateral upper extremity symptom distribution and headache, and severely limited, painful cervical spine range of motion including pain with passive flexion. These findings were deemed atypical for cervical radiculopathy by the examining chiropractor as they did not follow an expected unilateral dermatomal or myotomal pattern. The patient’s unusual clinical features prompted a cervical spine MRI, which ultimately revealed the diagnosis of CEH. To our knowledge, this case is the first report of a chiropractor identifying SEH. As the patient had no specific risk factors for SEH and diclofenac is not associated with SEH to our knowledge [14], it seems possible given other similar recent studies [5-8] that his COVID-19 illness could have been a predisposing factor for SEH.

As in the current case, patients with spontaneous SEH may have a normal neurologic examination [1]. Unfortunately, there is often a delay between symptom onset and onset of neurologic deficits, which can take several hours, days, or even months [1]. In one series of 20 patients with SEH, 40% of patients underwent MRI over 36 hours after symptom onset, while three patients (15%) underwent MRI over 30 days after symptom onset [15]. Diagnosis of CEH may be challenging and is based on the overall context of the patient’s history, the presence of risk factors, and examination findings [1]. Physical examination findings vary and may include one or more findings of diminished cervical spine range of motion, hypoesthesia, hypo- or hyperreflexia, pathological reflexes such as the Babinski sign, or upper or lower extremity weakness [1,7,16]. When spontaneous SEH is suspected, MRI is the optimal imaging modality [1].

The imaging features, in this case, were typical for CEH. The epidural mass was hyperintense on T1, mildly hyperintense on T2, and remained hyperintense on T1 fat-suppressed and T2 fat-suppressed sequences. According to its MRI signal, the epidural mass most likely represented a hematoma with a late subacute composition of intracellular methemoglobin and was most likely about one to two weeks old [17]. In comparison, an epidural abscess is almost always accompanied by meningeal thickening and rim enhancement with contrast [18]. Further, there was no evidence of discitis, spondylodiscitis, or paraspinal mass, any of which are typically also present in cases of epidural abscess [19]. A tumor was unlikely based on the imaging features as these are typically rim or heterogeneously enhancing with contrast [19]. While acute hematomas may show enhancement, these are generally non-enhancing as there is no blood supply [17,19]. Therefore, the absence of contrast enhancement, in this case, helped rule out abscess and tumor and rule in a hematoma.

According to a search of PubMed and Google Scholar on November 18, 2022, we identified four previously published cases of spontaneous SEH in patients following COVID-19 infection [5-8]. Several mechanisms have been suggested to explain these events, including venous thromboembolism, disseminated intravascular coagulation, increased pressure in the epidural venous plexus, and vasculitis [5-7], however, there is no clear unifying pathophysiological explanation. In addition, larger studies have identified an association between COVID-19 and other conditions involving bleeding such as intracranial hemorrhage and spontaneous muscle hematoma [9,10]. While evidence regarding the relationship between COVID-19 and SEH is limited, there may be an underlying bleeding mechanism.

While SEH is most often treated with surgery via decompressive hemilaminectomy or laminectomy [1], they may be treated non-operatively with a positive outcome [16,20]. A systematic review concluded that the decision towards either form of care should be based on the severity of neurological deficit and the clinical course [20], however, non-operative treatment is also recommended for patients who are asymptomatic or not surgical candidates [1]. Given the limited number of available cases, it is unknown if SEH related to COVID-19 has a better or worse prognosis with non-operative care compared to SEH related to other etiologies.

As CEH is a contraindication for spinal manipulative therapy [4], the most common treatment utilized by chiropractors [11], chiropractors must be aware of the urgency of recognizing this condition. Chiropractors should be vigilant to detect CEH in patients with neck pain having atypical, severe features, cases worsening despite conservative care, and those with risk factors for SEH. Further, chiropractors should refer patients with suspected CEH for urgent medical attention.

A larger study, potentially of a cross-sectional design, is needed to determine the prevalence of SEH in patients suffering from or recovering from COVID-19 and risk factors for this condition (e.g., COVID-19 severity, comorbidities, medications). Additional research is also needed to determine how often patients with SEH present to chiropractors, which could be accomplished using a large medical records dataset or practitioner survey.

## Conclusions

The present case highlights a patient with spontaneous CEH which occurred closely following recovery from COVID-19 and was identified by a chiropractor via MRI. Although COVID-19 appears to be a risk factor for other bleeding disorders, further research is needed to determine whether it is a risk factor for CEH. Clinicians such as chiropractors who manage spinal disorders must be vigilant to recognize clinical features or risk factors for CEH as this condition may be fatal. While CEH may be treated non-operatively with a positive outcome, referral for emergency care and/or neurosurgical evaluation is advised.
